# Case report: Comprehensive management of painful diabetic neuropathy—Addressing opioid-induced hyperalgesia through multimodal approaches

**DOI:** 10.1097/MD.0000000000039126

**Published:** 2024-08-02

**Authors:** Robert Maksymowicz, Cyprian N. Strączek, Jeremi J. Matysek, Dominika M. Lech, Krzysztof Nosek

**Affiliations:** a Department of Pharmacology and Toxicology Center for Experimental Medicine, Faculty of Medical Sciences, University of Warmia and Mazury, Olsztyn, Poland; b Pain Treatment Clinic, Provincial Specialist Hospital in Olsztyn, Olsztyn, Poland.

**Keywords:** case report, diabetic neuropathy, gabapentinoid, neuropathic pain, opioid-induced hyperalgesia

## Abstract

**Rationale:**

Diabetic neuropathy is a prevalent and debilitating complication of diabetes, necessitating effective pain management strategies. While pharmacological treatments, including opioids, are commonly employed, they pose significant challenges due to the risk of developing opioid-induced hyperalgesia (OIH). This case report aims to illustrate the efficacy of a comprehensive, multidisciplinary approach in managing painful diabetic neuropathy complicated by OIH.

**Patient concerns:**

A 64-year-old male patient presented to the Pain Treatment Clinic with severe lower limb pain due to diabetic polyneuropathy. He had a history of multiple comorbidities.

**Diagnoses:**

The patient’s condition and physical examination suggested the presence of opioid-induced hyperalgesia (OIH). Despite the increased dose of opioids, the patient did not report significant constipation or breathing difficulties but experienced drowsiness and dry mouth. A diagnosis of opioid and benzodiazepine dependence was made.

**Interventions:**

The treatment plan involved the initiation of pregabalin and duloxetine, gradual reduction of opioid use, and psychiatric support for addiction management.

**Outcomes:**

Over 12 months, the patient experienced significant pain reduction and minimal adverse effects.

**Lessons:**

Effective management of OIH involves gradual opioid tapering and a multimodal therapeutic approach. However, the optimal treatment strategies and the frequency of OIH occurrence remain areas of uncertainty, relying heavily on clinical expertise and individualized patient care. Further research is needed to refine these treatment strategies and improve patient outcomes.

## 1. Introduction

Diabetic neuropathy is a commonly occurring complication of diabetes. At the time of type 2 diabetes diagnosis, approximately 10% to 20% of patients concurrently present symptoms of peripheral neuropathy, with clinical manifestations of polyneuropathy occurring in 26% of patients after 5 years of disease duration. It is estimated that 50% to 66% of individuals with type 2 diabetes will develop peripheral neuropathy during their lifetime.^[1]^ Effective pain management plays a crucial role in the treatment of diabetic peripheral neuropathy, considering that approximately one-third of diabetic patients experience distressing symptoms such as spontaneous burning pain in the feet.^[2]^ Currently, the primary methods for managing painful diabetic neuropathy include strategies focused on glycemic control, lifestyle modifications, and the use of analgesic therapy.^[3]^ Pharmacological treatment includes the use of anticonvulsants, antidepressants, and analgesic medications, including opioid drugs, although opioids are not first-line treatment for painful diabetic neuropathy. A rare adverse effect of analgesic therapy may be opioid-induced hyperalgesia (OIH), occurring in <0.1% of patients treated with opioids for chronic pain.^[4]^

In this article, a case of a patient diagnosed with painful diabetic neuropathy, who developed symptoms of OIH, was presented.

## 2. Case presentation

A 64-year-old male patient with a history of severe lower limb pain due to severe diabetic polyneuropathy was presented to the Pain Treatment Clinic. His comorbidities included chronic pancreatitis, gastric ulcers, diabetic foot syndrome, hypertension, chronic gastritis, and liver steatosis. At the time of the first visit, the patient was taking estazolam 2 mg/day (for many years), Oxycodone 6 × 80 mg/day, Atorvastatin, Pantoprazole, Regular Human Insulin, Lispro Insulin, Lercanidipine, Ramipril, Cholecalciferol, calcium carbonate, and Kreon. The daily dose of oxycodone was 480 mg, as only such a high dose provided the patient with partial relief (numerical rating scale [NRS] 4–5/10). This dose had been gradually increased over the previous year due to uncontrolled pain. The patient condition and physical examination suggested the presence of OIH. Surprisingly, the patient did not report significant constipation or breathing difficulties but experienced drowsiness and dry mouth. A diagnosis of opioid and benzodiazepine dependence was made. A psychiatric consultation at the Addiction Treatment Clinic was recommended. The patient was also informed about other possible treatments for neuropathic pain.

During the consultation at the Pain Treatment Clinic, pregabalin was initiated at an initial dose of 1 × 75 mg/day, and duloxetine at a dose of 1 × 30 mg/day. Prophylactic use of laxatives was prescribed, and a gradual reduction in the doses of oxycodone and estazolam was recommended. Due to the established addiction, it was necessary to continue psychiatric and psychological care. During subsequent visits, due to an increase in creatinine levels (1.9 mg/dL), a decrease in Hb (9.6 g/dL), and hyperkalemia, the dose of angiotensin-converting enzyme inhibitor was reduced with the intention of discontinuation in the future. The patient was consulted by nephrologist and cardiologist. Chronic kidney disease was diagnosed. Abdominal ultrasound was performed, revealing no pathologies.

After 12 months of care at the Pain Treatment Clinic, the patient reported a significant reduction in baseline pain intensity (NRS 2-3/10). Benzodiazepine was discontinued, and the dose of oxycodone was reduced to 2 × 120 mg/day (from 6 × 80 mg/day). The dose of pregabalin was increased to 2 × 150 mg/day, and duloxetine to 1 × 60 mg/day. Peripheral edema and dizziness occurred to a minor extent after initiating the gabapentinoid, but these adverse effects were fully accepted by the patient.

## 3. Outcome

The case study presents a 64-year-old male patient with severe diabetic polyneuropathy and OIH who was successfully managed through a multidisciplinary approach. Initially, the patient experienced significant lower limb pain managed with a high dose of oxycodone (480 mg/day), resulting in partial pain relief but leading to the development of OIH. Over twelve months, a comprehensive treatment plan was implemented, involving the initiation of pregabalin and duloxetine, gradual opioid tapering, and psychiatric support for addiction management. The patient baseline pain intensity of 4 to 5/10 on the NRS decreased to 2 to 3/10 after 12 months, indicating a significant reduction in pain levels. The daily dose of oxycodone was successfully reduced from 480 mg (6 × 80 mg/day) to 240 mg (2 × 120 mg/day). Pregabalin was started at an initial dose of 75 mg/day and gradually increased to 300 mg/day (2 × 150 mg/day), while duloxetine was initiated at 30 mg/day and increased to 60 mg/day. This multimodal approach not only reduced the patient pain but also addressed the underlying opioid dependence, highlighting the effectiveness of individualized, comprehensive pain management strategies.

## 4. Discussion

OIH is characterized by the development of increased sensitivity to painful stimuli associated with chronic opioid therapy, rarely occurring during acute opioid therapy.^[5,6]^ It is a paradoxical phenomenon of lowering the pain threshold that may occur with escalating opioid doses. The central nervous system neuroplasticity, involving long-term potentiation (LTP) and neural circuit polarization (NCP), plays a leading role in the development of chronic pain and OIH.^[6,7]^ LTP is a neurobiological mechanism that governs the memory process. Several studies have indicated that LTP may also be responsible for maintaining chronic pain. The molecular mechanism of LTP in chronic pain involves the activation of the N-methyl-D-aspartate receptor (NMDAR) and the release of brain-derived neurotrophic factor (BDNF) by neurons and astrocytes in the spinal dorsal horn (SDH) and dorsal root ganglia.^[6,7]^

The pathomechanism of OIH is highly complex. The NMDA receptor can be stimulated by opioid drugs,^[8–10]^ and their binding activates protein kinase C, leading to receptor phosphorylation. Phosphorylation neutralizes the magnesium blockade, increasing calcium influx and, consequently, enhancing pain conductivity.^[9]^ Another mechanism contributing to opioid hyperalgesia is the desensitization of opioid receptors.^[10]^ The administration of opioid receptor agonists activates the synthesis of ganglioside GM1, causing the receptor to detach from the Gi/G0 protein. This protein is then replaced by the Gs protein. The opioid receptor, coupled with the Gs protein, stimulates pain conductivity instead of inhibiting it.^[9]^ Another mechanism is associated with the action of the endogenous opioid dynorphin A.^[8–10]^ This molecule is produced in the spinal cord and liver, and its release increases in prolonged pain and during treatment with exogenous opioids. Dynorphin may enhance the secretion of pronociceptive peptides, such as cholecystokinin and nociceptin-orphanin FQ.^[10]^

Descending control of pain impulses related to the activation of on-off cells in the ventrorostral part of medulla oblongata and the activation of GABAergic transmission in the midbrain are additional mechanisms in OIH development.^[10]^ The mechanisms underlying hyperalgesia provide the basis for pharmacological intervention using NMDAR antagonists such as ketamine and esketamine, which are used in preventing and treating OIH.^[6]^ A study by Liu X et al showed that morphine could alter the frequency and amplitude of excitatory postsynaptic currents in both excitatory and inhibitory neurons in the SDH.^[7]^ This polarization of postsynaptic currents may facilitate the development of OIH in the spinal dorsal horn. This phenomenon is known as NCP. These 2 facilitative mechanisms in OIH may lead to the formation of an endogenous hyperactive neuronal pacemaker with pathological activation of sodium and calcium channels, effectively treated with antiepileptic drugs such as gabapentin, pregabalin, or phenytoin.^[5]^ In the case of our patient, the chronic and gradually increasing administration of oxycodone led to the development of OIH through the mechanisms described above.

Chronic activation of serotonin receptor type 2 (5-HT2) can also lead to OIH. Studies indicate that administering 5-HT2 antagonists in buprenorphine-induced hyperalgesia resulted in reduced activation of astrocytes and reversal of buprenorphine-nduced hyperalgesia.^[6]^ Jiang S et al demonstrated in their case report that administering duloxetine to a lung cancer patient with severe pain significantly reduced the amount of opioids required for pain relief and completely reversed OIH.^[11]^

The treatment of neuropathic pain poses a significant challenge. Only 50% of individuals with painful diabetic polyneuropathy respond to monotherapy. Therefore, in patients with only a partial response to a single drug or in whom monotherapy at high doses cannot be used due to side effects, combination therapy is necessary.^[2]^ However, effective treatment is achieved in only one-third of patients.^[1]^ Currently recommended treatment for painful diabetic peripheral neuropathy (DPN) has largely remained unchanged over the last decade. Drugs with the most confirmed efficacy in treating painful DPN include pregabalin and gabapentin, ligands for the α2δ subunit of voltage-gated calcium channels, as well as duloxetine, a serotonin and norepinephrine reuptake inhibitor (SNRI). In addition to these drugs, opioid medications such as buprenorphine, oxycodone, morphine, tapentadol (norepinephrine reuptake inhibitor), and tramadol (non-opioid mechanism of action - inhibition of norepinephrine and serotonin reuptake) are used in neuropathic pain therapy. Tricyclic antidepressants, such as amitriptyline, lidocaine patches, and botulinum toxin, are also used. Some new therapies have received local approvals for use in painful DPN, such as the α2δ ligand mirogabaline (in Japan) and the 8% capsaicin patch (approved by the Federal Drug Administration and in Europe).^[2,3]^

## 5. Conclusions

In the treatment of neuropathic pain caused by diabetic neuropathy, drugs from the gabapentinoid group (pregabalin and gabapentin) and norepinephrine and serotonin reuptake inhibitors (duloxetine) are preferred due to their more specific mechanism of action and less troublesome side effects.^[3]^ In our patient, the combination therapy of pregabalin at a dose of 2 × 150 mg/day and duloxetine at a dose of 1 × 60 mg/day was well-tolerated and effective (NRS 2–3).

Caution should be exercised when using opioids (excluding tramadol and tapentadol) for neuropathic pain due to the lack of clear data regarding their efficacy, concerns about addiction, and potential adverse effects (such as OIH).^[3]^ The aforementioned side effects occurred in the presented case.

Constipation is a common side effect of opioid therapy for pain. It is a significant complication of such therapy and can substantially impact patient adherence and quality of life. To improve adherence to medical recommendations and enhance the quality of life, any long-term opioid therapy should be accompanied by concurrent administration of a laxative.^[12,13]^

A rare but troublesome side effect of opioid therapy is OIH. Escalation of opioid doses can lead to the induction of this adverse effect; therefore, it is crucial to monitor the effectiveness and side effects of opioid analgesic drugs. Monitoring allows for modifications in pain management (e.g., adding a coanalgesic). In the case of OIH, the physician should individualize the therapy for the specific patient. Gradual dose reduction of the used opioid, opioid rotation, and the use of multimodal therapy (combining opioid analgesic, non-opioid analgesic, and coanalgesic) are employed in the management.^[14]^ The rate of opioid dose de-escalation should be around 10% to 20% of the initial dose every 4 weeks (slow taper) or weekly (fast taper).^[15]^ The gradual reduction of oxycodone dosage and the inclusion of pregabalin and duloxetine in the pain management strategy led to a reduction in pain and facilitated the gradual discontinuation of opioids in our patients.

Currently, it is challenging to find publications that provide an exact frequency of OIH occurrence, and different described treatment strategies are difficult to compare. The therapy of OIH significantly relies on the physician own experience and individual decisions.

## 6. Limitations

The study has several limitations, including the subjectivity of pain measurement, which relies on patient-reported outcomes like the NRS that can vary significantly between individuals. The patient multiple comorbidities, such as chronic pancreatitis, gastric ulcers, diabetic foot syndrome, hypertension, chronic gastritis, and liver steatosis, could have influenced symptom presentation and treatment response, complicating the interpretation of the treatment effectiveness. Additionally, the patient was on a complex medication regimen, making it challenging to isolate the effects of the treatment specifically aimed at managing OIH. The follow-up period was limited to twelve months, and a longer follow-up is needed to understand the long-term efficacy and safety of the multimodal approach used. The treatment plan, involving gradual opioid reduction, initiation of pregabalin and duloxetine, and psychiatric support, was tailored to the patient specific needs, limiting the ability to standardize and replicate the protocol in other settings. The success of the treatment heavily relied on the clinicians’ expertise and experience, which may not be easily replicable in different clinical environments with varying levels of expertise. Furthermore, the patient opioid and benzodiazepine dependence required ongoing psychiatric and psychological care, adding complexity to the management and highlighting the intricate interaction between psychological factors and pain perception. These limitations underscore the need for further research, including larger cohort studies and randomized controlled trials, to better understand and validate the multimodal treatment strategies for managing painful diabetic neuropathy and OIH.

## Author contributions

**Conceptualization:** Robert Maksymowicz, Cyprian Norbert Strączek, Jeremi Jan Matysek, Dominika Maja Lech, Krzysztof Nosek.

**Funding acquisition:** Krzysztof Nosek.

**Project administration:** Robert Maksymowicz, Cyprian Norbert Strączek, Jeremi Jan Matysek, Dominika Maja Lech.

**Supervision:** Krzysztof Nosek.

**Writing** – **original draft:** Robert Maksymowicz, Cyprian Norbert Strączek, Jeremi Jan Matysek, Dominika Maja Lech.

**Writing – review & editing:** Robert Maksymowicz, Cyprian Norbert Strączek, Jeremi Jan Matysek, Dominika Maja Lech, Krzysztof Nosek.
